# Hypolipidemic and Anti-Inflammatory Effects of *Curcuma longa*-Derived Bisacurone in High-Fat Diet-Fed Mice

**DOI:** 10.3390/ijms24119366

**Published:** 2023-05-27

**Authors:** Chaoqi He, Taiki Miyazawa, Chizumi Abe, Takahiro Ueno, Mikiko Suzuki, Masashi Mizukami, Kazue Kurihara, Masako Toda

**Affiliations:** 1 Laboratory of Food and Biomolecular Science, Graduate School of Agricultural Science, Tohoku University, Sendai 980-8572, Japan; he.chaoqi.p5@dc.tohoku.ac.jp (C.H.);; 2 Food Biotechnology Platform Promoting Project, New Industry Creation Hatchery Center (NICHe), Tohoku University, Sendai 980-8579, Japan; taiki.miyazawa.b3@tohoku.ac.jp (T.M.); chizumi.abe.e5@tohoku.ac.jp (C.A.); 3 Center for Radioisotope Sciences, Tohoku University Graduate School of Medicine, Sendai 980-8575, Japan; suzukimikiko@med.tohoku.ac.jp; 4 New Industry Creation Hatchery Center (NICHe), Tohoku University, Sendai 980-8579, Japan; masashi.mizukami.e7@tohoku.ac.jp (M.M.); kazue.kurihara.b7@tohoku.ac.jp (K.K.)

**Keywords:** bisacurone, high-fat diet, cholesterol, blood viscosity, pro-inflammatory cytokines, NF-κB pathway

## Abstract

Turmeric (*Curcuma longa*) contains various compounds that potentially improve health. Bisacurone is a turmeric-derived compound but has been less studied compared to other compounds, such as curcumin. In this study, we aimed to evaluate the anti-inflammatory and lipid-lowering effects of bisacurone in high-fat diet (HFD)-fed mice. Mice were fed HFD to induce lipidemia and orally administered bisacurone daily for two weeks. Bisacurone reduced liver weight, serum cholesterol and triglyceride levels, and blood viscosity in mice. Splenocytes from bisacurone-treated mice produced lower levels of the pro-inflammatory cytokines IL-6 and TNF-α upon stimulation with a toll-like receptor (TLR) 4 ligand, lipopolysaccharide (LPS), and TLR1/2 ligand, Pam3CSK4, than those from untreated mice. Bisacurone also inhibited LPS-induced IL-6 and TNF-α production in the murine macrophage cell line, RAW264.7. Western blot analysis revealed that bisacurone inhibited the phosphorylation of IKKα/β and NF-κB p65 subunit, but not of the mitogen-activated protein kinases, p38 kinase and p42/44 kinases, and c-Jun N-terminal kinase in the cells. Collectively, these results suggest that bisacurone has the potential to reduce serum lipid levels and blood viscosity in mice with high-fat diet-induced lipidemia and modulate inflammation via inhibition of NF-κB-mediated pathways.

## 1. Introduction

Turmeric (*Curcuma longa*) has been widely used as a food spice and attracted interest as a source of compounds with antitumor, anti-inflammatory, and lipid-lowering effects [1,2,3,4]. To date, approximately 235 compounds have been isolated from *C. longa*, most of which are phenolic compounds and terpenoids [5]. Among them, curcumin is considered the most active constituent of *C. longa*. The health benefits of curcumin have been extensively assessed for the prevention and treatment of inflammatory diseases [6,7], whereas those of other compounds in *C. longa* have not been extensively investigated.

Bisacurone, a phytochemical present in *C. longa*, is a bisabolane-type sesquiterpenoid consisting of three isoprene-derived units [8]. Bisacurone inhibits TNF-α-mediated soluble vascular cell adhesion molecule-1 (VCAM-1) expression in inflammatory monocytes and cancer cells [9]. Feeding mice with bisacurone reduced total lipid, triglyceride, and cholesterol levels in the liver [10]. *C. longa* extracts containing bisacurone suppress the development of ethanol-induced liver injury [11,12] and non-alcoholic fatty liver disease (NAFLD) [13] in murine models by reducing serum levels of pro-inflammatory cytokines, such as IL-6 and TNF-α, and improving liver weight, fat accumulation, and levels of biochemical markers, such as serum cholesterol and aspartate aminotransferase (AST)/alanine aminotransferase (ALT). A randomized, double-blind intervention trial involving middle-aged and elderly subjects with overweight or mild hypertension showed that a hot water extract of *C. longa* containing bisacurone decreased the serum levels of C-reactive protein, IL-6, TNF-α, and VCAM-1 [14].

The expression of inflammatory markers is increased in secondary lymphoid organs, in addition to liver and adipose tissues, in diet-induced obese mice [15,16]. However, the effects of bisacurone on the pro-inflammatory responses of immune cells in the lymphoid tissues remain unclear. In the present study, we aimed to assess the hypolipidemic and anti-inflammatory effects of bisacurone in high-fat diet (HFD)-fed mice. In addition, we attempted to elucidate the mechanism responsible for the anti-inflammatory effect of bisacurone using a murine macrophage cell line RAW264.7. This study provides insights into the use of bisacurone as a hypolipidemic and anti-inflammatory molecule for the development of functional foods.

## 2. Results

### 2.1. Bisacurone Reduces Serum Levels of Lipids and Blood Viscosity in HFD-Fed Mice

**Figure 1:**
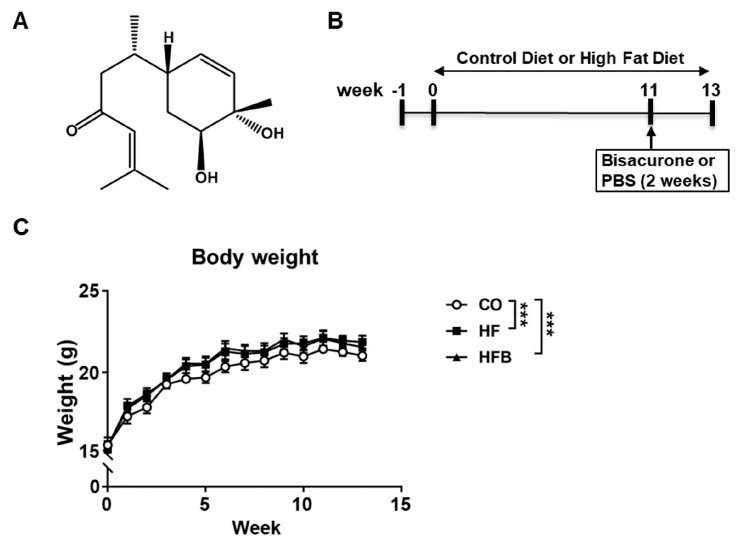
(**A**) The structural formula of bisacurone. (**B**) Treatment schedule of bisacurone. After adaptation for one week, BALB/c mice received a control diet or a high-fat diet for eleven weeks. HFD-fed mice were orally administered with bisacurone in PBS (HFB group), or PBS only (HF group) every day for two weeks. Control diet-fed mice were orally administered with PBS (CO group). (**C**) The influence of bisacurone on body weight in HFD-fed mice. Data represent the mean ± SEM of two independent experiments. *** p < 0.001, two-way ANOVA, Tukey’s multiple comparisons test.

**Figure 2:**
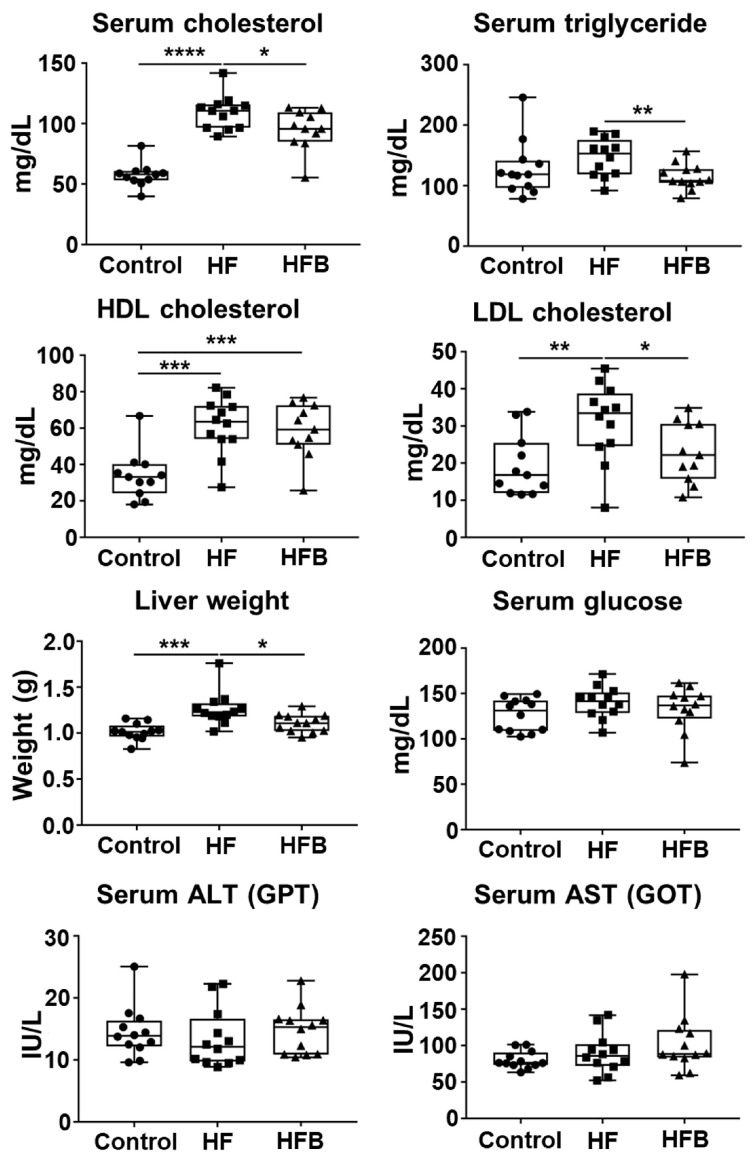
The influence of bisacurone on the serum levels of biochemical parameters in HFD-fed mice. BALB/c mice (*n* = 6/group) received an HFD or a control diet. HFD-fed mice were orally administered with bisacurone in PBS or PBS only. Control diet-fed mice received oral administration of PBS. The weight of livers and the levels of total, LDL, HDL cholesterols, triglyceride, glucose, aspartate aminotransferase (AST), and alanine aminotransferase (ALT) in sera from the mice were measured. Data represent the mean ± SEM of two independent experiments. Each symbol indicates an individual mouse. Control: Control diet-fed and PBS-treated group. HF: HFD-fed and PBS-treated group. HFB: HFD-fed and bisacurone-treated group. * *p <* 0.05, ** *p <* 0.01, *** *p <* 0.001, **** *p <* 0.0001, student’s *t*-test.

First, we assessed the effect of bisacurone on glycemia and lipidemia in mice on an HFD. The structural formula of bisacurone is shown in Figure 1A. BALB/c mice received HFD for 11 weeks and were orally administered bisacurone for two weeks daily (Figure 1B). The HFD increased serum levels of total, HDL, and LDL cholesterols and triglyceride and weights of the liver and whole body of mice compared to a control diet (Figure 1C and Figure 2), while bisacurone reduced the serum levels of total and LDL cholesterols and triglyceride and the weight of the livers of HFD-fed mice (Figure 2). Serum levels of HDL cholesterol, AST and ALT were not significantly different between bisacurone-treated and untreated HFD-fed mice (Figure 2). Bisacurone did not influence body weight (Figure 1C) and serum glucose levels in the mice (Figure 2).

**Figure 3:**
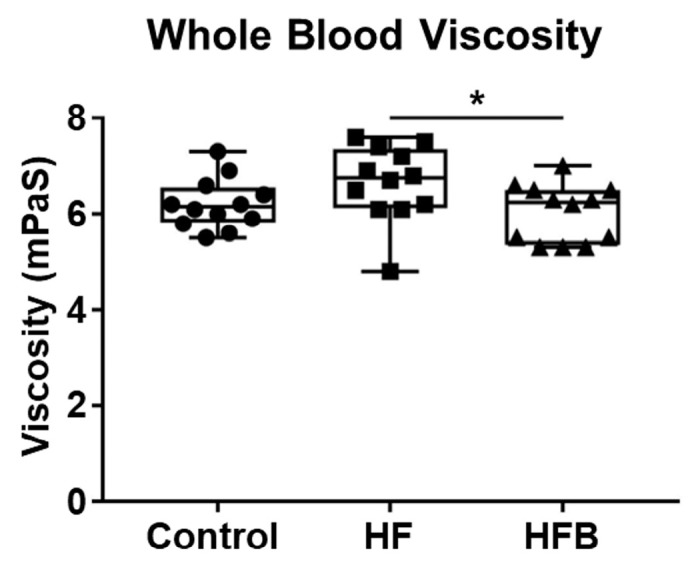
The influence of bisacurone on the blood viscosity of HFD-fed mice. BALB/c mice (*n* = 6/group) received an HFD or a control diet. HFD-fed mice were orally administered with bisacurone in PBS, or PBS only. Control diet-fed mice were orally administered with PBS. The viscosity of blood samples harvested from the mice was measured using an ultra-micro viscometer. Each symbol indicates an individual mouse. Data represent the mean ± SEM of two independent experiments. Control: Control diet-fed and PBS-treated group. HF: HFD-fed and PBS-treated group. HFB: HFD-fed and bisacurone-treated group. * *p <* 0.05, student’s *t*-test.

Previous studies have shown that blood viscosity is affected by serum lipid levels [17,18,19,20]. Increased blood viscosity has been observed in several diseases, including hyperlipidemia [17] and metabolic syndromes [18]. Therefore, we measured the viscosity of blood from HFD-fed and bisacurone-treated mice and control mice. The viscosity of blood from mice on the HFD for 11 weeks was higher than that from mice on a control diet. The viscosity of bisacurone-treated mice on the HFD was lower than that of phosphate-buffered saline (PBS)-treated mice on the same diet (Figure 3). The results showed that bisacurone reduced lipidemia and blood viscosity in HFD-fed mice.

**Figure 4:**
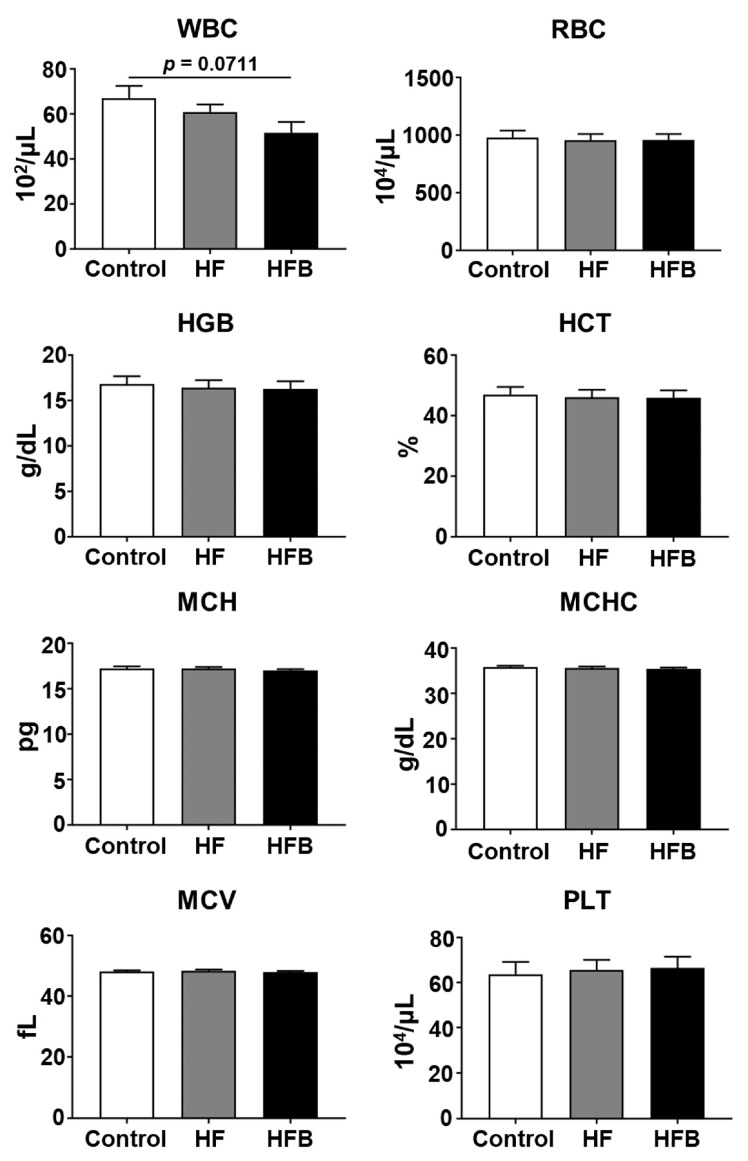
The influence of bisacurone on hematological parameters in HFD-fed mice. BALB/c mice (*n* = 10) received an HFD or a control diet. HFD-fed mice were orally administered with bisacurone in PBS or only PBS. Control diet-fed mice were orally administered with PBS. Blood was harvested from mice, and hematological parameters were measured. Data represent the Mean ± SEM, student’s *t*-test. WBC: White blood cell count; RBC: Red blood cell count; HGB: Hemoglobin; HCT: Hematocrit; MCV: Mean corpuscular volume; MCH: Mean corpuscular hemoglobin; MCHC: Mean corpuscular hemoglobin concentration; PLT: Platelet count. Control: Control diet-fed and PBS-treated group. HF: HFD-fed and PBS-treated group. HFB: HFD-fed and bisacurone-treated group.

Next, we analyzed the effect of bisacurone on the following hematological parameters: white blood cell (WBC) count, red blood cell (RBC) count, hemoglobin (HGB), hematocrit (HCT), mean corpuscular volume (MCV), mean corpuscular hemoglobin (MCH), mean corpuscular hemoglobin concentration (MCHC), and platelet (PLT) count. Although the levels of the tested hematological parameters were not significantly different among control diet-fed and PBS-treated group, HFD-fed and PBS-treated group, and HFD-fed and bisacurone-treated group, there was a decreasing trend in the WBC count of the bisacurone-treated mice (Figure 4). These results suggest that bisacurone does not influence hematological parameters, except WBC, in the HFD-fed mice.

### 2.2. Bisacurone Reduces the Production of Pro-Inflammatory Cytokines in Spleens of HFD-Fed Mice

**Figure 5:**
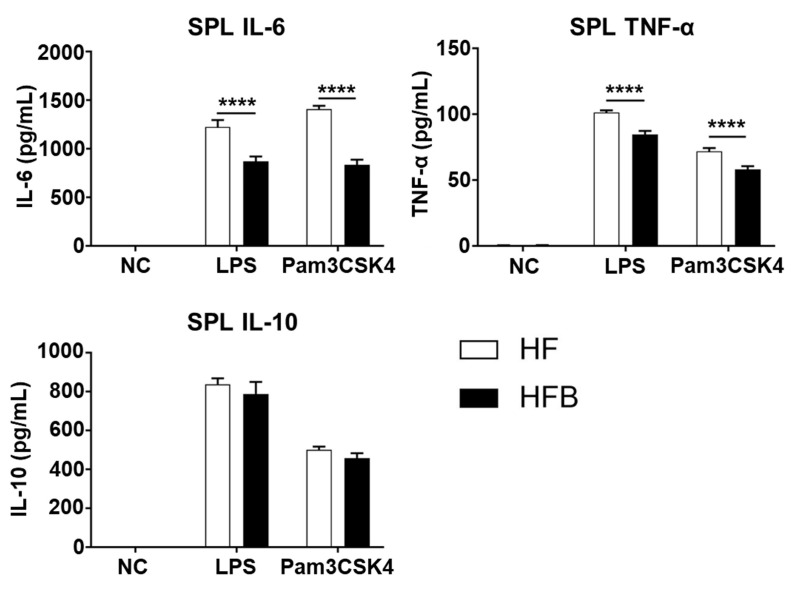
The influence of bisacurone on the production of pro-inflammatory cytokines in spleens of HFD-fed mice. The splenocytes were prepared at 2 × 10^6^ cells/mL (*n* = 6) and stimulated with LPS (1 µg/mL) or Pam3CSK4 (5 µg/mL) for 24 h. The concentrations of IL-6, TNF-α, IL-1β and IL-10 in the culture supernatants were measured by ELISA. Data represent the mean ± SEM of two independent experiments. HF: HFD-fed and PBS-treated group. HFB: HFD-fed and bisacurone-treated group. **** *p* < 0.0001, student’s *t*-test.

Next, to determine whether oral administration of bisacurone influences pro-inflammatory responses in the spleen, the major secondary lymphoid organ, under an HFD condition, splenocytes from the bisacurone-, or PBS-treated mice on the HFD were stimulated with LPS, a TLR4 ligand, or Pam3CSK4, a TLR2 ligand. The concentrations of pro-inflammatory cytokines IL-6 and TNF-α were significantly lower in the culture supernatants of splenocytes from bisacurone-treated mice, compared to those from PBS-treated mice (Figure 5). The concentration of IL-1β in the supernatants of splenocytes was not detected. The concentrations of the anti-inflammatory cytokine IL-10 in the supernatants of splenocytes from bisacurone-treated and PBS-treated mice were not significantly different.

**Figure 6:**
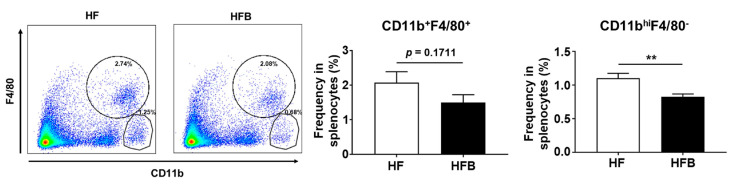
The influence of bisacurone on the frequency of splenocytes in HFD-fed mice. The frequency of macrophages in spleens of bisacurone-treated or non-treated mice (*n* = 6) on HFD was measured by FACS. Representative dot blot data (left) of CD11b and F4/80 expressing cells in the spleens and the frequency of splenocytes (right) expressing CD11b and/or F4/80 were shown. Data represent the mean ± SEM of two independent experiments. HF: HFD-fed and PBS-treated group. HFB: HFD-fed and bisacurone-treated group. ** *p* < 0.01, student’s *t*-test.

A source of IL-6 and TNF-α in TLR2 ligand- or TLR4 ligand-stimulated splenocytes is macrophages. Next, we assessed the frequency of macrophages in the mouse spleens. Fluorescence-activated cell sorting (FACS) analysis showed that the frequency of macrophage populations CD11b^+^ F4/80^+^ and CD11b^high^F4/80^−^ was reduced in bisacurone-treated mice, compared to PBS-treated mice (Figure 6). These results suggest that the oral administration of bisacurone influences the frequency of macrophage populations in the spleen.

### 2.3. Bisacurone Inhibits Cytokine Production in RAW264.7 Cells

Bisacurone reduced the frequency of macrophages in mouse spleens. It was unclear whether the reduced production of IL-6 and TNF-α in LPS- and Pam3CSK4-stimulated splenocytes from bisacurone-treated mice was owing to reduced frequency of macrophages or involvement of signaling pathways associated with cytokine expression in the cells. Therefore, we performed in vitro experiments using a macrophage cell line RAW264.7 to assess whether bisacurone influences cytokine production and its associated pathways in the cells.

First, we assayed cytotoxicity by adding CCK-8 solution after stimulating RAW264.7 cells with different concentrations of bisacurone and LPS for 24 h. The results showed that RAW264.7 cells were not cytotoxic under co-stimulation with 50 μM bisacurone and 10 ng/mL LPS (Figure S1A). Flow cytometry results of 7-amino actinomycin D (7-AAD) staining showed that bisacurone did not increase the number of dead cells up to 50 μM in the presence of LPS stimulation (Figure S1B).

**Figure 7:**
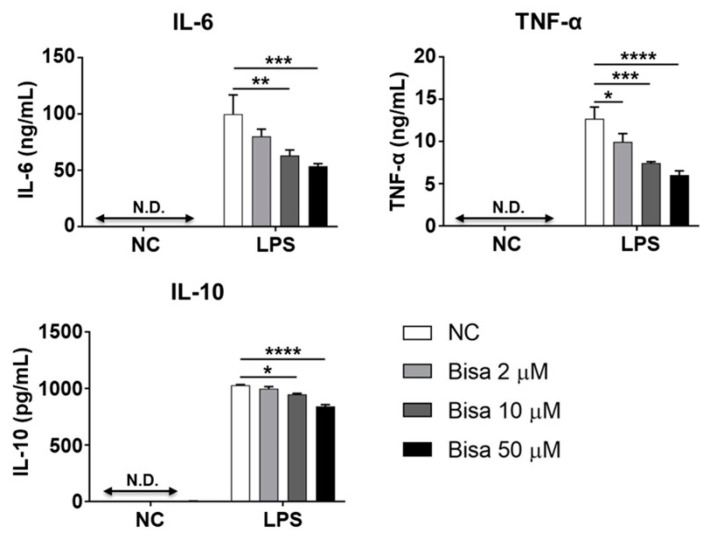
The influence of bisacurone on cytokine production in a murine macrophage line. RAW264.7 cells were prepared at 1 × 10^6^ cells/mL (*n* = 3) and treated with 2, 10, or 50 μM bisacurone and stimulated with 10 ng/mL of LPS for 24 h. The concentrations of IL-6, TNF-α, and IL-10 in the culture supernatants were measured by ELISA. Data shows the mean ± SEM and represent three independent experiments. * *p* < 0.05, ** *p* < 0.01, *** *p* < 0.001, **** *p* < 0.0001. Ordinary one-way ANOVA, Tukey’s multiple comparisons test. Bisa: Bisacurone. N.D.: not detectable.

LPS stimulation induced detectable levels of IL-6, IL-10, and TNF-α in RAW264.7 cells (Figure 7). Pretreatment with bisacurone inhibited the production of these cytokines in LPS-stimulated cells, although reduction in level of IL-10 was low (Figure 7). Previous studies showed that the NF-κB and MAP kinase pathways are involved in the induction of IL-6, IL-10, and TNF-α expression [21,22], whereas the STAT3 pathway is mostly involved in the induction of IL-6 and IL-10 [23]. To elucidate the mechanism underlying the inhibitory effect of bisacurone on cytokine production in RAW264.7 cells, western blotting was performed to assess the activation of proteins in these pathways.

**Figure 8:**
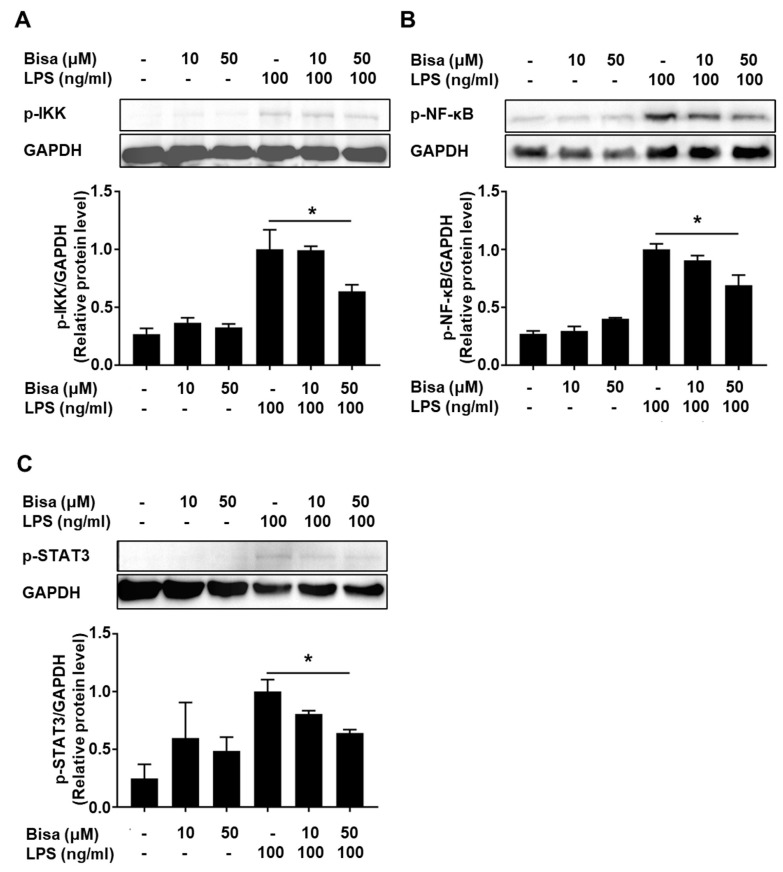
The influence of bisacurone on activation of NF-κB cascade and STAT3 in a murine macrophage strain. RAW264.7 cells were treated with 10 or 50 μM bisacurone and stimulated with 100 ng/mL of LPS for 30 min or 6 h. Phosphorylation of (**A**) IKKα/β, (**B**) NF-κB p65 subunit and (**C**) STAT3, together with GAPDH, in the cells were detected by western blotting. Data represent the mean ± SEM of three independent experiments. * *p <* 0.05. Ordinary one-way ANOVA, Tukey’s multiple comparisons test. Bisa: Bisacurone.

**Figure 9:**
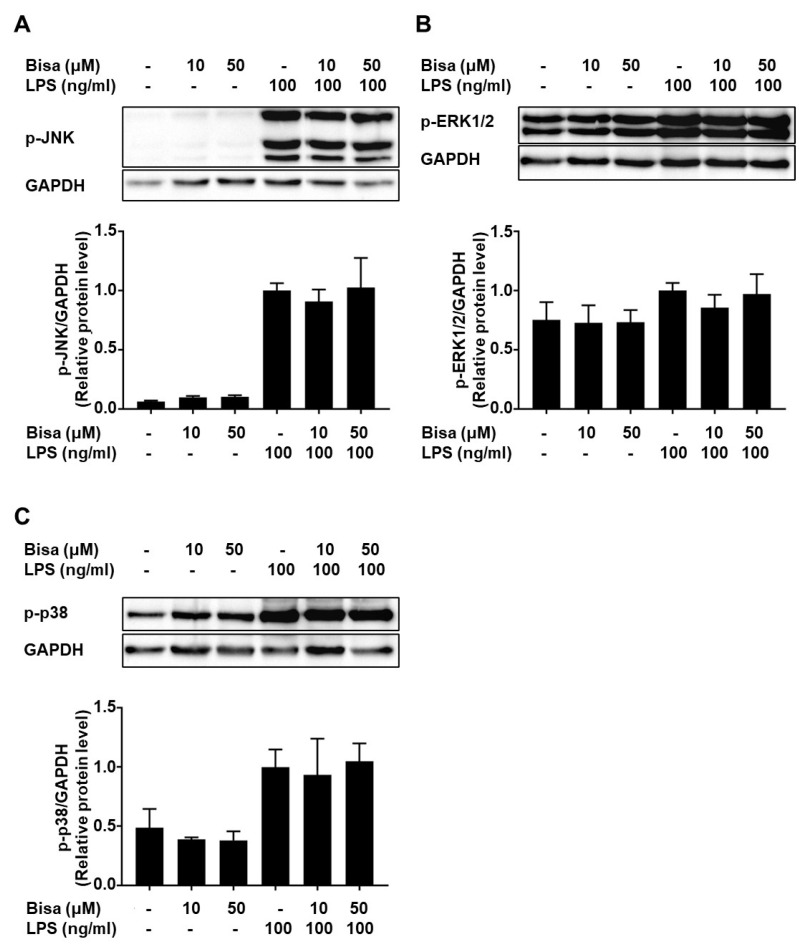
The influence of bisacurone on activation of MAP kinases in a murine macrophage strain. RAW264.7 cells were treated with 10 or 50 μM bisacurone and stimulated with 100 ng/mL of LPS for 30 min. (**A**) Phosphorylated JNK, (**B**) ERK1/2, and (**C**) p38 kinase in the cells, together with GAPDH, was detected by western blotting. Data represent the mean ± SEM of three independent experiments. Ordinary one-way ANOVA, Tukey’s multiple comparisons test. Bisa: Bisacurone.

Phosphorylation of IκB kinase (IKK) α/β and NF-κB p65 subunit was detected 30 min after LPS stimulation, whereas bisacurone reduced the phosphorylation (Figure 8A,B). Phosphorylation of STAT3 was not detected at 30 min but was detected at 6 h after LPS stimulation; however, bisacurone reversed this effect (Figure 8C). Similarly, phosphorylation of ERK1/2, JNK, and p38 kinase in the cells was detected 30 min after LPS stimulation. Bisacurone did not significantly alter the phosphorylation of these three MAP kinases (Figure 9A–C). These results suggest that bisacurone affects the NF-κB and STAT3 pathways, but not the MAP kinase pathway in RAW264.7 cells.

Several studies have shown that antioxidants can modulate cellular signaling events [24,25]. Therefore, we assessed the antioxidant capacity of bisacurone using a 2,2-Diphenyl-1-picrylhydrazyl (DPPH) assay [26]. Trolox, the water-soluble vitamin E analogue and a conventional antioxidant standard, generates DPPH-H by quenching the DPPH radicals. When the antioxidant capacity of Trolox, a vitamin E analogue, was set to 1.0, those of bisacurone and a well-known antioxidant, curcumin, were 0.294 ± 0.001 and 0.900 ± 0.087, respectively, indicating that the antioxidant capacity of bisacurone is lower than curcumin. These results suggest that the inhibitory effect of bisacurone on the NF-κB pathway would not be due to the antioxidant effect. 

## 3. Discussion

In this study, we showed that bisacurone inhibited production of the pro-inflammatory cytokines IL-6 and TNF-α in LPS-stimulated macrophages. Moreover, bisacurone inhibited the phosphorylation of IKKα/β and NF-κB p65 subunit, which are involved in the activation of NF-κB pathway. The NF-κB pathway plays a central role in the production of pro-inflammatory factors, including IL-6 and TNF-α, in immune cells. Our results suggest that bisacurone exhibits anti-inflammatory effects by inhibiting the NF-κB pathway, which is consistent with a previous study reporting the inhibitory properties of 18 types of bisabolane-type sesquiterpenoids extracted from turmeric, including bisacurone C (an isomer of bisacurone used in this study) on the expression of TNF-α, IL-6, and IL-8, at mRNA and protein levels in A549 cells, a human lung carcinoma with properties of type II alveolar epithelial cells [27]. Sun et al. showed that bisacurone inhibits the phosphorylation of IκBα and reduces the adhesion of TNF-α-activated human umbilical vein endothelial cells via VCAM-1 downregulation [9]. The NF-κB pathway is initiated by the IKK activation via IKKα/β phosphorylation. Activated IKK catalyzes the phosphorylation of IκBα, which results in the activation of IκB kinase and the subsequent phosphorylation of the NF-κB p50/p65 heterodimer for nuclear translocation and gene expression of pro-inflammatory factors. Our study suggests that bisacurone interferes with the NF-κB pathway by inhibiting phosphorylation of IKKα/β.

Several polyphenols inhibit the NF-kB cascade, which has been explained by their capacity to interact with IKKs and IκB directly, or to reduce generation of reactive oxidation species (ROS) [28,29,30]. Reactive oxidation species (ROS) are generated by various cellular processes as part of cellular signaling events, and antioxidants reduce ROS generation. In the NF-κB pathway, ROS are involved in various steps, such as enhancing IKKα/β and IκBα phosphorylation [24,28]. Considering its low antioxidant capacity, bisacurone may inhibit the phosphorylation of IKK α/β and NF-κB p65 by direct interaction with signaling molecule(s), but not by reduction in ROS activity. Bisacurone also inhibited the production of anti-inflammatory cytokine IL-10 in LPS-stimulated RAW264.7 cells. The result suggests that IL-10 is not a molecule mediating the anti-inflammatory effect of bisacurone. Signaling mechanism of TLR4-induced IL-10 synthesis is divided into two phases: synthesis via NF-κB cascade and JAK1/STAT3 cascade [31,32]. Initially, IL-10 synthesis is induced via the NF-κB cascade. IL-10 synthesized in the initial phase then binds to IL-10 receptor in an autocrine and paracrine manner, that triggers JAK1/STAT3 cascade and additional IL-10 synthesis. Notably, bisacurone reduced the phosphorylation of NF-κB p65 unit and STAT3. Bisacurone would inhibit initial IL-10 synthesis by inhibiting NF-κB cascade, which reduces IL-10 receptor-mediated phosphorylation of STAT3 and subsequent synthesis of IL-10. 

Oral administration of bisacurone reduced the production of IL-6 and TNF-α in TLR2- and TLR4-stimulated splenocytes from the HFD-fed mice. This may be owing to the suppressive effect of bisacurone on the frequency of macrophages in mouse spleens. Curcumin and other polyphenols reduce macrophage accumulation in the liver and adipose tissue by elevating adiponectin levels [33]. Obesity and lipidemia promote chronic inflammation in multiple tissues including the liver and spleen. Accumulation of pro-inflammatory macrophages has been observed in the white adipose tissues of HFD-fed rodents and patients with obesity and dyslipidemia [34,35,36,37]. Saturated and oxidized fatty acids act as TLR4 ligands and induce the accumulation of pro-inflammatory macrophages by expressing pro-inflammatory chemokines [35]. Our results suggest that bisacurone ameliorates tissue inflammation during lipidaemia. In addition, bisacurone inhibits TLR4-mediated cell activation by inhibiting of NF-κB cascade. The hypolipidemic and inhibitory effects of bisacurone on NF-κB cascade might reduce the frequency of macrophage population in the spleens of HFD-fed mice.

Bisacurone exhibited a lowering effect on blood cholesterol and triglycerides and reduced the size of the liver in HFD-fed mice. This is consistent with a previous study reporting that extracts of *C. longa* containing bisacurone reduce blood cholesterol levels in a murine model of NAFLD [13]. Ashida et al. showed that bisacurone inhibits lipid synthesis by regulating gene expressions involved in lipid homeostasis. Bisacurone enhances the expression of liver kinase B1 (LKB1), peroxisome proliferator-activated receptor α (PPARα), and carnitine palmitoyl transferase 1 (CPT-1), but decreases the expression of sterol response element binding protein (SREBP1), CCAAT/enhancer binding protein (C/EBP) α, and carbohydrate response element binding protein (ChREBP) [10]. 

Bisacurone also reduced the serum levels of LDL cholesterol, but not HDL cholesterol in HFD-fed mice. Yang et al. showed that oral administration of 50 μg/kg bisacurone reduced the serum levels of LDL cholesterol and increased HDL cholesterol in diabetic rats [38]. The discrepancy may be explained by use of different animal species. Luo et al. showed that polyphenols stimulate low-density lipoprotein receptor-mediated uptake of LDL cholesterol by activating SREBP2 and PPARδ [39]. It is necessary to investigate whether bisacurone reduces the levels of LDL cholesterol by a molecular mechanism similar to that of polyphenols.

In this study, bisacurone was also found to reduce blood viscosity in HFD-fed mice. The factors influencing blood viscosity include hematocrit, cholesterol, triglycerides, and the number of RBCs, WBCs, and platelets. Bisacurone reduced the levels of cholesterol and triglycerides, but did not alter the levels of hematocrit and platelets, and tended to reduce the number of WBCs only slightly. However, the direct association between blood lipids (cholesterol or triglycerides) and viscosity remains debatable. Turpin et al. showed that high blood lipid levels induce membrane peroxidation or increase the cell membrane rigidity of RBCs, thereby altering the blood viscosity [40]. The reduced blood viscosity by bisacurone could be attributed to the hypolipidemic effect of the molecule; however, the detailed mechanism needs to be elucidated further. 

In conclusion, we demonstrated that bisacurone exsert hypolipidemic and anti-inflammatory effects. The hypolipidemic effect of bisacurone would lead to a reduction in blood viscosity and macrophage-associated inflammatory responses in tissues. The anti-inflammatory effect of bisacurone could be partly explained by its inhibitory effect on the NF-κB cascade. Further in vivo studies elucidating the detailed mechanisms underlying the hypolipidemic and anti-inflammatory effects of bisacurone are warranted to elucidate its application in functional foods for health benefits. A limitation of our study was that the effects of bisacurone were assessed only under HFD conditions and not under normal diet conditions. It is also crucial to determine whether and how bisacurone influences biochemical parameters and blood viscosity under normal conditions in order to develop better foods.

## 4. Materials and Methods

### 4.1. Bisacurone Reduces Serum Levels of Lipids and Blood Viscosity in HFD-Fed Mice

BALB/c mice (female, 6 weeks old) were purchased from CLEA Japan (Shizuoka, Japan). After adaptation for one week, the mice received a HFD (30 kcal% fat, Quick fat diet, CLEA Japan), or a control diet (12 kcal% fat, CE-2 diet, a recommended control for Quick fat diet by CLEA Japan) for eleven weeks and oral administration of 50 μg of bisacurone (Nagara Science Co., Ltd., Gifu, Japan) in PBS or PBS only using a gastric needle every day for two weeks. The dose of bisacurone was based on previous studies assessing the effect of bisacurone on hepatic lipid accumulation and diabetes nephropathy in rodent models [10,38]. The composition of Quick fat diet and CE-2 is indicated in Table S1. 

The mice were housed under pathogen-free conditions. All animal experiments were performed in accordance with the rules on animal experiments at Tohoku University (2022AgA-017). The mice were maintained under standard conditions (room temperature:22 ± 2 °C; relative humidity: 55 ± 10%) on a light and dark cycle of 12:12 h of artificial light (lights on from 8 A.M. to 8 P.M.). At the end of the experiment, mice were euthanized by cervical decapitation. 

### 4.2. Measurement of Blood Viscosity

The mice were sacrificed, and the blood samples were collected in tubes containing 0.5 M EDTA (Nacalai Tesque Inc. Kyoto, Japan) as an anticoagulant [41]. Blood viscosity was measured by an ultra-trace viscometer RSM-MV1, which was invented by Kurihara and her group at NICHe of TOHOKU University, Japan. This device is currently commercialized by SMILEco Instruments Co., Ltd., (Sendai, Japan).

### 4.3. Measurement of Serum Biochemical Parameters

Serum levels of biochemical parameters, glucose, cholesterol, triglyceride, and AST/ALT were measured using LabAssay^TM^ Glucose, LabAssay^TM^ Cholesterol, LabAssay^TM^ Triglyceride, and Transaminase CII-Test Wako. These kits were from FUJI Film Wako Shibayagi Cooperation (Shibukawa, Japan). Serum levels of HDL and LDL cholesterols were measured using HDL and LDL/VLDL Quantitation Kit (MAK045, Sigma-Aldrich, St. Louis, MO, USA).

### 4.4. Measurement of Hematological Parameters

Hematological parameters including red blood cell count, white blood cell count, hemoglobin concentration, platelet count in blood samples were measured by Auto Blood Cell Counter MEK-6450 (Nihon-Kohden, Shinjuku, Japan). The hematocrit, mean corpuscular volume, mean corpuscular hemoglobin, and mean corpuscular hemoglobin concentration were calculated from the measured data above described.

### 4.5. Splenocyte Assay

Spleens were harvested from the mice after two weeks of oral administration of bisacurone. Splenocytes were prepared and stimulated at 2 × 10^6^ cells/mL with LPS from *Salmonella typhimurium* (1 µg/mL: Sigma-Aldrich) or Pam3CSK4 (5 µg/mL: FUJIFILM Wako Pure Chemical Corporation, Osaka, Japan) for 24 h. The concentrations of cytokines in cell culture supernatants were measured by ELISA, using primary and secondary monoclonal antibody (mAb) sets (clone MP5-20F3 and clone MP5-32C11) in combination with streptavidin- horseradish peroxidase (HRPO) for IL-6 detection, ELISA MAX™ Standard Set Mouse TNF-α, ELISA MAX™ Standard Set Mouse IL-1β, and ELISA MAX™ Standard Set Mouse IL-10. Monoclonal Abs and ELISA kits were purchased from BioLegend (San Diego, CA, USA).

### 4.6. FACS Analysis

Splenocytes (2 × 10^6^ cells) were incubated with either monoclonal antibody (mAb) against CD16/CD32 (clone 2.4G2, Thermo Fisher Scientific, Waltham, MA, USA), or normal murine IgG antibodies (Santa Cruz Biotechnology, Dallas, TX, USA) for treatment of IgG receptors on the cell surface, and stained with allophycocyanin (APC) anti-mouse F4/80 mAb (clone QA17A29) and phycoerythrin (PE) anti-mouse/human CD11b mAb (clone M1/70) together with 7-AAD viability staining solution. Monoclonal Abs and 7-AAD were from BioLegend. Fluorescence intensity of the cells in 7-AAD negative population (viable cells) was measured by FACSLyric (BD Bioscience, Franklin Lakes, NJ, USA).

### 4.7. Cellular Assay

Raw264.7 cells were obtained from Riken BioResource Research Center (Tsukuba, Japan), and maintained in Minimum Essential Media (MEM: FUJIFILM Wako Pure Chemical Corporation) containing 10% fetal bovine serum (Sigma-Aldrich), 0.1 mM non-essential amino acids (FUJIFILM Wako Pure Chemical Corporation), 100 units/mL of penicillin, and 100 μg/mL of streptomycin (FUJIFILM Wako Pure Chemical Corporation). For cytokine production assay, the cells (1 × 10^6^ cells/mL) were stimulated with 10 ng/mL of LPS in the presence or absence with 2 or 10 μM bisacurone for 24 h. The concentrations of IL-6, IL-10, and TNF-α in the cell culture supernatants were measured by ELISA as described in Section 4.5. 

### 4.8. Western Blot Analysis

Raw 264.7 cells (1 × 10^6^ cells/mL) were stimulated with 100 ng/mL of LPS in the presence or absence of 10 or 50 μM bisacurone for 30 min, or for 6 h [42]. After incubation, the cells were lysed in lysis buffer based on 62.5 mM Tris-HCl (pH 6.8) containing 2% *w*/*v* SDS, 10% glycerol, 50 mM dithiothreitol, 0.01% *w*/*v* bromophenol blue, DNase (Worthington Biochemical, Lakewood, NJ, USA) and RNase (R6513, Sigma-Aldrich). Aliquots of 15 μL were resolved by 12.5% SDS-PAGE and transferred to Amersham™ Hybond™ (Cytiva: Marlborough, MA, USA). After blocking with 3% bovine serum albumin (FUJIFILM Wako Pure Chemical Corporation) for 1 h, membranes were incubated with following primary antibodies specific for phosphorylated-IKK (2694), (p)-NF-kB-p65 subunit (Ser536) (3033), p-JNK (4668), p-ERK1/2 (4370), p-p38 kinase (4511), or p-STAT3 (9145) from Cell Signaling Technology, Inc., (Danvers, MA, USA), at 4 °C overnight. The membranes were then incubated with and HRP-linked anti-rabbit IgG (Cell Signaling Technology, Inc.) and HRP-conjugated GAPDH (Proteintech Group, Inc., Rosemont, IL, USA) at room temperature for 1 h, and developed using Chemi-Lumi One Ultra (Nacalai Tesque). The signals were visualized using WSE-6100 LuminoGraph I (ATTO Corporation, Tokyo, Japan). The intensities of protein bands were quantitated by ImageJ software v1.52a (www.imagej.nih.gov, accessed on 1 August 2020, National Institutes of Health, Bethesda, MD, USA).

### 4.9. DPPH Antioxidant Assay

The antioxidant property of bisacurone was assessed using DPPH Antioxidant Assay Kit (D678, DOJINDO Laboratories, Kumamoto, Japan). Briefly, DPPH working solution was incubated with Trolox (2.5 to 80 μg/mL) or bisacurone (0.8 to 100 μg/mL). The radical scavenging effects of test molecules in generating DPPH-H (colorless molecule) were assessed by measuring the levels of the remaining DPPH in the test solutions at 517 nm.

### 4.10. Statistical Analysis

Results were expressed as mean ± SEM. Statistical significance was determined using two-tailed unpaired-Student’s *t* test or one-way analysis of variance (ANOVA), Tukey’s multiple comparisons test with a single pooled variance. A difference was considered significant at *p* < 0.05.

## Data Availability

The data that support the findings of this study are available from the corresponding authors, upon reasonable request.

## Supplementary Materials

The following supporting information can be downloaded at: https://www.mdpi.com/article/10.3390/ijms24119366/s1.
