# Supplementary material for: Hypolipidemic and Anti-Inflammatory Effects of Curcuma longa-Derived Bisacurone in High-Fat Diet-Fed Mice
Source: Int J Mol Sci. 2023 May 27;24(11):9366. doi: 10.3390/ijms24119366 (PMC10253162; doi:10.3390/ijms24119366)
Supplement: Supplementary file 1 [file ijms-24-09366-s001.zip › ijms-2349171-supplementary.pdf]

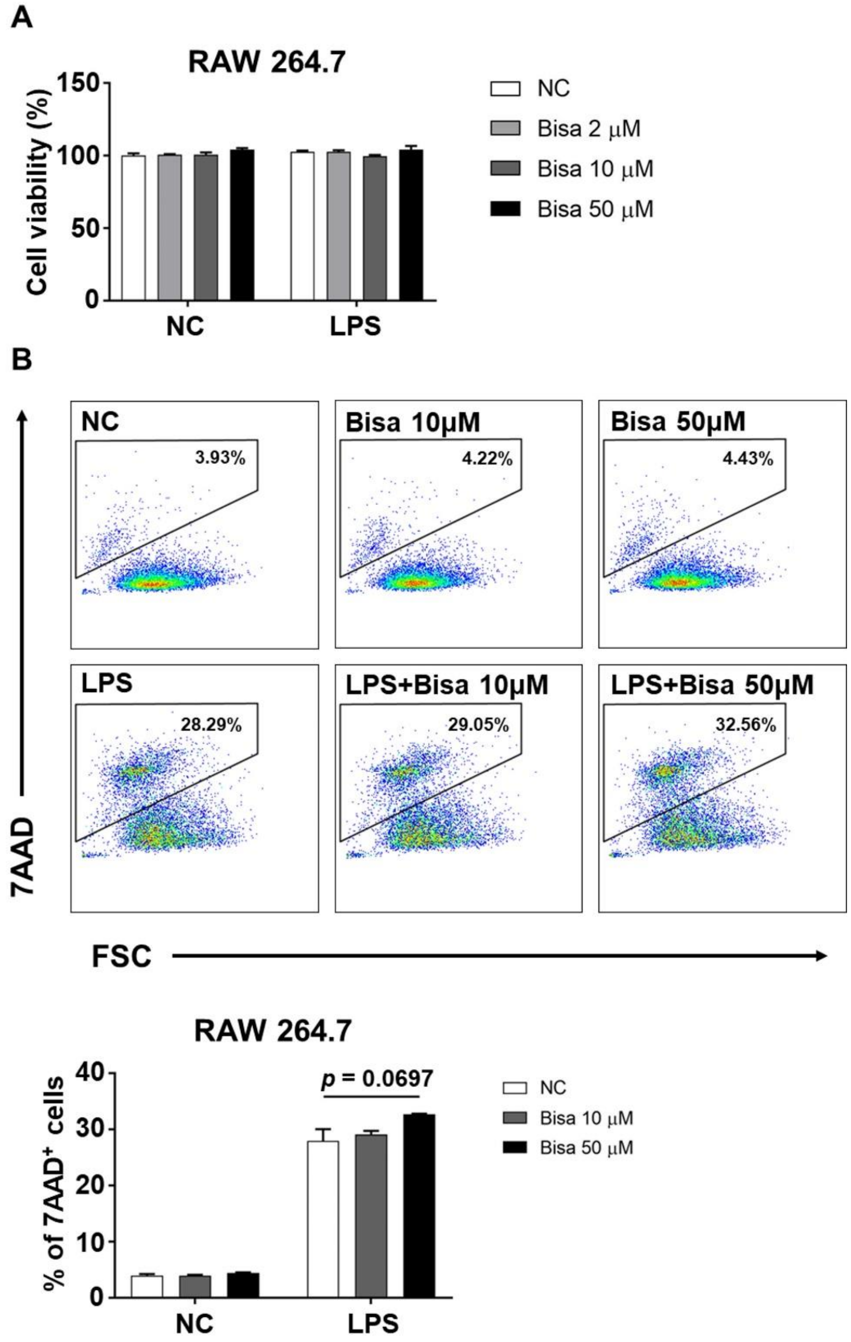

Figure S1 . Assessment for the cytotoxicity of bisacurone in a murine macrophage line. RAW264.7 cells were treated with 2, 10, or 50 μM bisacurone and stimulated with 10 ng/mL of LPS for 24 hours. For assessment of cell viability, (A) the concentrations of lactate dehydrogenase in cell culture supernatants were measured using Cell Counting Kit-8 (DOJINDO LABORATORIES, Japan). (B) The cells were harvested and stained with 7-Amino-Actinomycin D (7AAD) for FACS analysis. Data represent the mean ± SEM. Ordinary one-way ANOVA, Tukey's multiple comparisons test. Bisa: bisacurone

Table S1. Composition of diets used in this study*.

	Control diet (CE-2)		High fat diet (Quick fat)	
Ingredients	gm%	kcal%	gm%	kcal%
Moisture (%)	8.88	0	6.80	0
Crude protein (%)	25.05	29	24.35	24
Crude fat (%)	4.77	12	13.80	30
Crude fiber (%)	4.44	0	3.00	0
Crude ash(%)	7.04	0	5.75	0
NFE (%)	49.82	59	46.30	46
Energy (kcal/100g)	342.4		406.80	

Ingredients	Control diet (CE-2)	High fat diet (Quick fat)
Protein	whitefish meal, soybean meal, yeast	soybean meal, whitefish meal, casein, yeast
Fat	cereal germ, soybean oil	animal fat and oil (beef tallow), cereal germ
Carbohydrates	wheat flour, corn, milo	wheat flour, corn, millet

*Data from URL of CLEA Japan, Inc.

https://www.clea-japan.com/en/products/general_diet/item_d0030

https://www.clea-japan.com/en/products/general_diet/item_d0070
